# Case Report: Potential of pharmacological treatment for auditory abnormal sensations with aripiprazole: a report of two cases

**DOI:** 10.3389/fpsyt.2025.1614500

**Published:** 2025-06-03

**Authors:** Fumiyuki Goto, Shoji Kaneda, Kenji Okami, Koichiro Wasano

**Affiliations:** Tokai University Isehara Hospital, Isehara, Japan

**Keywords:** auditory abnormal sensations, aripiprazole, pseudo-hallucinations, phonophobia, central sensory processing dysfunction

## Abstract

Auditory abnormal sensations encompass various subjective auditory symptoms such as tinnitus, hyperacusis, aural fullness, autophony, dysacusis, pseudo-hallucinations, and misophonia. Although tinnitus management typically includes lifestyle counseling and sound therapy, there remains no established treatment for symptoms like aural fullness or pseudo-hallucinations with unknown etiology. In cases where central sensory processing abnormalities or emotional instability are suspected, psychotropic medications may offer benefit. We report two cases in which aripiprazole, an atypical antipsychotic, was effective in treating such symptoms. In both cases, traditional approaches such as antidepressants and supportive therapy were insufficient, but aripiprazole led to marked improvement in subjective auditory symptoms. These cases suggest a potential role for pharmacological modulation of central sensory and emotional regulation in patients with auditory abnormal sensations. One involved a man with phonophobia and aural fullness, and the other a woman with tinnitus and pseudo-hallucinations.

## Introduction

Auditory abnormal sensations refer to a spectrum of subjective auditory complaints that are not fully explained by conventional otologic or neurologic findings. These sensations include tinnitus, hyperacusis, aural fullness, autophony, dysacusis, pseudo-hallucinations, and misophonia. While tinnitus is relatively well-studied and often managed through sound therapy and counseling strategies such as tinnitus retraining therapy (TRT) or cognitive behavioral therapy (CBT) (1, 2), other auditory symptoms remain poorly understood and lack established therapeutic options.

Auditory abnormal sensations, including tinnitus, aural fullness, and hyperacusis, are reported in approximately 10–15% of the population, with 1–2% experiencing severe functional impairment. These symptoms are associated with insomnia, anxiety, depression, and social withdrawal, resulting in a significant reduction in quality of life (QOL) (3, 4).

Patients with auditory abnormal sensations often present with significant distress and functional impairment despite normal or near-normal audiological assessments. In such cases, central sensory processing dysfunction and emotional dysregulation are increasingly recognized as contributing factors (4, 5). These central mechanisms may lead to heightened auditory perception, altered affective responses to sound, or even the misinterpretation of internally generated auditory stimuli as external sounds, such as in pseudo-hallucinations (6, 7).

Psychotropic agents, including selective serotonin reuptake inhibitors (SSRIs), serotonin-norepinephrine reuptake inhibitors (SNRIs), and benzodiazepines, have been applied in various neurotologic disorders, especially when comorbid anxiety or depression is evident. More recently, low-dose aripiprazole, an atypical antipsychotic with partial dopamine D2 receptor agonist activity and serotonin receptor modulation, has shown promise in the treatment of refractory dizziness and related sensory-emotional disorders (8, 9). These findings suggest that modulation of central neurochemical pathways may alleviate symptoms stemming from sensory-emotional dysregulation.

However, the use of aripiprazole in treating auditory abnormal sensations—particularly in cases without frank psychosis—has not been well documented. Given the overlapping pathophysiology between chronic dizziness and perceptual auditory disturbances, there is a rationale for exploring aripiprazole’s utility in this context. We herein report two cases of auditory abnormal sensations—one presenting with aural fullness and phonophobia, and the other with tinnitus and pseudo-hallucinations—successfully treated with low-dose aripiprazole. These cases highlight the potential role of dopaminergic-serotonergic modulation in managing non-psychotic auditory perceptual abnormalities and provide preliminary support for pharmacologic intervention targeting central sensory processing. The sociodemographic and clinical details of these cases are summarized in **Table 1**.

**Table 1:** Sociodemographic and clinical characteristics of the cases.

Case	Age/Sex	Main Symptoms	Psychological Background (HADS)	Clinical Background/Medical History
Case 1	40s/Male	Aural fullness, Phonophobia	HADS-A: 10, HADS-D: 9	No psychiatric or neurological history; symptoms began after loud noise exposure
Case 2	73/Female	Tinnitus, Pseudo-hallucinations	HADS-A: 11, HADS-D: 12	History of migraine since youth; no prior psychiatric treatment

## Case 1

A man in his 40s developed aural fullness and phonophobia in his left ear after someone shouted loudly near it. The symptoms significantly interfered with his daily life, prompting him to visit a local clinic. The diagnostic assessments including pure-tone audiometry, tympanometry, speech discrimination testing, and head MRI were performed to exclude organic otological pathology. Pure tone audiometry revealed normal hearing, but he was initially diagnosed with acute sensorineural hearing loss and treated with corticosteroids, with no improvement. He was then referred to our department for further evaluation.

The severity of subjective symptoms was assessed using the Numerical Rating Scale (NRS), which ranges from 0 (no symptoms) to 10 (most severe). Generally, scores of ≤4 are considered mild, 5–6 moderate, and ≥7 severe symptom-related misperceptions (10).

The Hospital Anxiety and Depression Scale (HADS) (11) indicated mild anxiety (score 10) and mild depression (score 9). He was initially prescribed etizolam 0.5 mg/day, which led to mild symptomatic relief of both aural fullness and phonophobia (NRS 10 → 8) after two weeks. Due to his depressive tendency, vortioxetine 25 mg/day was subsequently initiated and maintained for three weeks, but no clinically significant change in auditory symptoms was observed. The patient also reported mild nausea and chose to discontinue antidepressants. Therefore, aripiprazole 1.5 mg/day was introduced as monotherapy. Two weeks later, both phonophobia and aural fullness improved (NRS 10 → 4), and at the 8-week follow-up, symptoms further decreased to NRS scores of 3.

## Case 2

**Figure 1:**
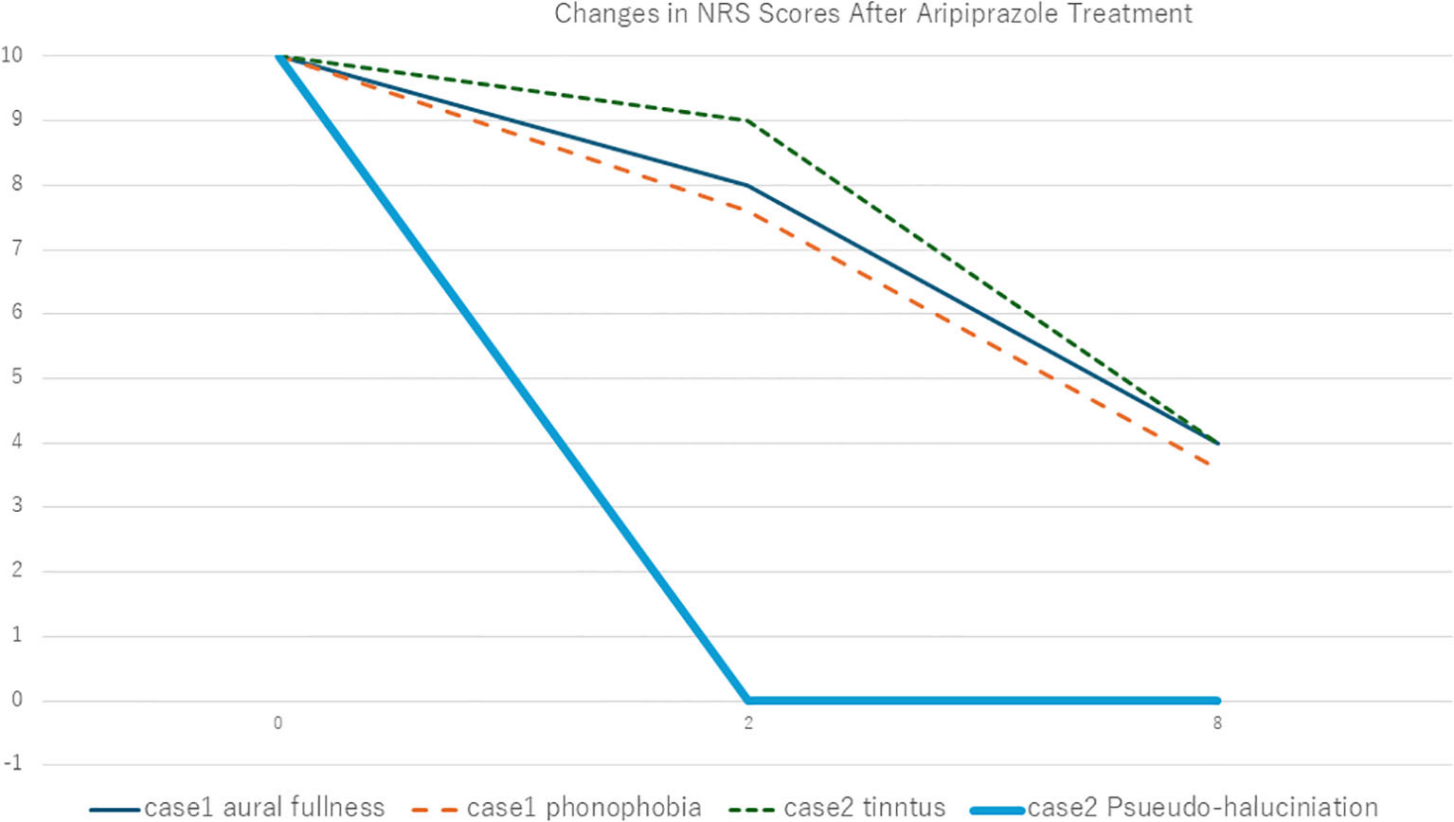
Changes in Numerical Rating Scale (NRS) scores following aripiprazole treatment in Case 1 and Case 2. Vertical axis is Numerical Rating Scale (NRS) and horizontal axis is weeks after treatment initiation.

A 73-year-old woman with a history of migraine since youth began experiencing tinnitus and pseudo-hallucinations in December of Year X. On presentation to our clinic, audiometry revealed low-frequency sensorineural hearing loss (see **Figure 1**). The diagnostic assessments including pure-tone audiometry, tympanometry, speech discrimination testing, and head MRI were performed to exclude organic otological pathology. Her Tinnitus Handicap Inventory (THI) score was 62, and her HADS scores were 11 for anxiety and 12 for depression.

Despite receiving lifestyle counseling—including avoidance of silence and active redirection of attention—neither her tinnitus nor pseudo-hallucinations improved. She was then started on low-dose amitriptyline (10 mg/day) and clonazepam (0.25 mg/day), which led to only modest subjective relief, with NRS scores remaining unchanged at 9 for both symptoms after two weeks. In pursuit of greater improvement, we initiated augmentation therapy with aripiprazole 1.5 mg/day. Two weeks later, her pseudo-hallucinations had fully resolved (NRS 0), while her tinnitus persisted (NRS 9). By the 8-week follow-up, her tinnitus also showed moderate improvement, with the NRS score decreasing to 4.

## Discussion

The present report highlights two cases of auditory abnormal sensations that responded favorably to low-dose aripiprazole after conventional treatment approaches had failed. The common pathophysiological thread between both cases appears to be central sensory hypersensitivity and impaired emotional regulation. These mechanisms are well documented in the context of tinnitus and hyperacusis, where dysfunctional neural networks involving the auditory cortex, limbic system, and prefrontal regions contribute to symptom persistence and distress (6, 7).

Aripiprazole is a dopamine system stabilizer that acts as a partial agonist at dopamine D2 receptors and modulates serotonergic pathways through 5-HT1A agonism and 5-HT2A antagonism (12). Originally developed as an antipsychotic, it has also shown efficacy in non-psychotic conditions such as functional dizziness and somatoform disorders (6, 8, 9). These effects may reflect its ability to regulate sensory gating, emotional reactivity, and cognitive appraisal of internal stimuli—mechanisms relevant to symptoms like pseudo-hallucinations and phonophobia.

In Case 1, the patient’s symptoms of phonophobia and aural fullness were resistant to corticosteroids, benzodiazepines, and even vortioxetine, an antidepressant with multimodal serotonergic action. The clear symptomatic reduction following aripiprazole introduction suggests that dopaminergic modulation played a key role in attenuating central hypersensitivity. In Case 2, pseudo-hallucinations unresponsive to low-dose tricyclic and benzodiazepine treatment were completely resolved with aripiprazole. This supports a potential role for atypical antipsychotics even in subclinical perceptual disturbances.

Aripiprazole may be particularly effective in patients whose auditory symptoms are thought to be associated with emotional dysregulation, including anxiety, depressive tendencies, or somatic symptom-related cognitive distortion. This aligns with its known dopaminergic-serotonergic modulatory action, which may reduce sensory hyperreactivity and maladaptive appraisal of internal stimuli (12). In contrast, aripiprazole is unlikely to be effective in cases where symptoms are due to identifiable organic causes, such as cochlear damage, retrocochlear lesions, or drug-induced auditory dysfunction. Careful case selection is therefore essential to optimize outcomes and avoid unnecessary pharmacological burden.

Importantly, neither patient exhibited frank psychotic features, and both tolerated aripiprazole without significant adverse effects. This expands the possible clinical use of aripiprazole into domains of non-psychotic sensory dysfunction. Nevertheless, these are preliminary observations, and placebo effects or spontaneous improvement cannot be ruled out. Controlled trials are necessary to validate these findings.

## Limitations and future directions

This report presents two cases suggesting the potential efficacy of aripiprazole in treating auditory abnormal sensations. However, several limitations should be acknowledged. First, as a case report involving only two patients, the findings are insufficient to generalize the effectiveness of this medication. Second, the evaluation of subjective symptoms was based on the Numerical Rating Scale (NRS), which, while convenient, is inherently subjective and susceptible to inter-rater variability and placebo effects. Third, the possibility of spontaneous symptom improvement cannot be entirely ruled out. Although the therapeutic effect of aripiprazole is suggested by the lack of response to prior medications, definitive conclusions require longer-term observation and controlled studies.

Furthermore, the neurophysiological mechanisms by which aripiprazole may affect sensory processing abnormalities and emotional regulation remain unclear. Comparative studies with other psychotropic agents are needed to clarify the specificity and relative efficacy of aripiprazole. Although no adverse effects were observed in either case, especially in elderly patients, drug sensitivity must be considered, and future studies should address the safety profile of aripiprazole in this population.

In light of these limitations, it is necessary to accumulate a larger number of clinical cases and to conduct prospective studies using standardized evaluation tools and objective indices. Such research will help establish the efficacy and safety of pharmacological treatment for auditory abnormal sensations in a more systematic manner.

In complex sensory-emotional cases involving auditory symptoms, collaboration with clinical pharmacists may help identify medication-related side effects or interactions, as seen in reports of drug-induced hallucinations (e.g., solifenacin, trimethoprim-sulfamethoxazole) (13, 14).

## Conclusion

In the absence of standardized treatments for auditory abnormal sensations, aripiprazole may represent a promising pharmacological option by targeting central sensory processing and emotional regulation. Further clinical studies are warranted to establish its efficacy and broaden its indications in otologic psychosomatic disorders.

## Data Availability

The raw data supporting the conclusions of this article will be made available by the authors, without undue reservation.
